# 

**Published:** 1888-03

**Authors:** 


### American Medical Association.

The Thirty-ninth Annual Session will be held in Cincinnati, Ohio, on Tuesday, Wednesday, Thursday, and Friday, May 8th, 9th, 10th, and 11th, commencing on Tuesday at 11 A. M.

"The delegates shall receive their appointment from permanently organized State Medical Societies, and such county and district Medical Societies *as are recognized by representation in their respective State Societies*, and from the Medical Department of the Army and Navy, and the Marine Hospital Service of the United States.

"Each State, County, and District Medical Society entitled to representation shall have the privilege of sending to the Association one delegate for every ten of its regular resident members and one for every additional fraction of more than half that number: *Provided*, however, that the number of delegates for any particular State, territory, county, city or town shall not exceed the ratio of one in ten of the resident physicians who may have signed the Code of Ethics of the Association.

Secretaries of Medical Societies, as above designated, are earnestly requested to forward, *at once*, lists of their delegates.

Also, that the permanent Secretary may be enabled to erase from the roll the names of those who have forfeited their membership, the Secretaries *are by special resolution*, requested to send to him, annually, a corrected list of the membership of their respective Societies.

*Sections.*—"The Chairman of each Section shall prepare an address on the recent advancements in the branches belonging to his Section, including such suggestions in regard to improvements in methods of work, and present, on the first day of its annual meeting, the same to the Section over which he presides. The reading of such address not to occupy more than forty minutes.

Practice of Medicine, Materia Medica and Physiology: Dr. ———, Chairman; Dr. N. S. Davis, Jr., 65 Randolph St., Chicago, Ill., Secretary.

Obstetrics and Diseases of Women and Children: Dr. Eli Van De Warker, 45 Montgomery St., Syracuse, N. Y., Chairman; Dr. E. W. Cushing, 1 Hotel, Pelham, Boston, Mass., Secretary.

Surgery and Anatomy: Dr. Donald McLean, 72 Lafayette Avenue, Detroit, Mich., Chairman; Dr. B. A. Watson, 124 York St., Jersey City, N. J., Secretary.

State Medicine: Dr. H. B. Baker, Lansing, Mich., Chairman; Dr. S. T. Armstrong, U. S. M. Hosp, Service Secretary.

Ophthalmology, Otology and Laryngology: Dr. F. C. Hotz, 181 Clark St., Chicago, Ill., Chairman; Dr. Edw. Jackson, 215 S. 17th St., Philadelphia, Pa., Secretary.

Diseases of Children: Dr. F. E. Waxham, 3449 Indiana Ave., Chicago, Ill., Chairman; Dr. W. B. Lawrence, Batesville, Ark., Secretary.

Oral and Dental Surgery: Dr. J. Taft, Cincinnati, Ohio, Chairman; Dr. E. S. Talbot, 125 State St., Chicago, Ill., Secretary.

Medical Jurisprudence: Dr. E. M. Reid, 243 N. Fremont St., Baltimore, Md., Chairman; Dr. C. B. Bell, Suffolk, Mass., Secretary.

Dermatology and Syphilography: Dr. L. D. Bulkley, 4 E. 37th St., New York, Chairman; Dr. S. F. Dunlap, Danville, Ky., Secretary.

A member desiring to read a paper before a Section should forward the paper, or its *title* and *length* (not to exceed twenty minutes in reading), to the Chairman of the Committee of Arrangements at least one month before the meeting.—*By-Laws.*

Committee of Arrangements.—W. W. Dawson, Cincinnati, Ohio, Chairman.

WM. B. ATKINSON, M.D.,
Permanent Secretary.

Philadelphia, 1400 Pine St., S. W. cor. Broad.

### EDITORIAL.

### Michigan State Dental Society.

The Thirty-third annual meeting of this veteran society will be held at the Dental College in Ann Arbor, beginning Tuesday, March 20th, at 10 o'clock, and continuing till Friday following.

This promises to be an exceedingly interesting meeting. The Executive Committee will have a very full and attractive programme. Quite a number of prominent members of the profession in neighboring States have promised to be present, and this will certainly add interest to the occasion. A number of papers have already been promised by those whom it will be a pleasure to hear.

Part of the time will be devoted to clinics and demonstrations, by some of the best operators in the country, The facilities for this work is all that could be desired, as large clinic rooms well supplied with all needed facilities are there ready, and also a good mechanical laboratory. The special and general museums connected with the University are available for illustration and demonstration. Some matters other than those of merely a practical nature will probably be brought before the meeting for discussion. The consideration of educational and ethical questions claim and should receive attention in the meetings of our State societies.

It is hoped that all will come prepared to make the meeting one of profit as well as of pleasure. Let every one be present at the beginning and stay till the end.

### Removal.

Drs. Bogue & Co., American Dentists in Paris, have removed their office from No. 39 Boulevard Haussmann to No. 73 Boulevard Haussmann.

This note will be of interest to their many friends on this side of the water, who may wish to refer parties to them, and of such there is a large number. It very frequently happens that dentists in this country, and especially in our large cities, are called on for reference to reliable practitioners in European countries, and it is always gratifying to be able to answer such requests satisfactorily.

It would be well if every dentist would keep a list with correct reference of reliable dentists in foreign countries, for this purpose. Reference of this kind is always very gratifying to those who have occasion to ask for it.

### CONTENTS.

COMMUNICATIONS.

Removal .......... 160

---

### THE DENTAL REGISTER,

**A MONTHLY MAGAZINE DEVOTED TO THE INTERESTS OF THE**

### DENTAL PROFESSION.

*Terms Reduced to* $2.00 *Per Year. Single Copies,* 25 *Cents.*

**EDITED BY PROF. J. TAFT, D.D.S.**

—AND—

**Published by SAM'L A. CROCKER & CO., Ohio Dental and Surgical Depot.**

The volume commences in January and closes in December.

The REGISTER being issued monthly is always fresh, therefore Indispensable to the practitioner who desires to keep posted up with the new inventions and improvements which are daily developed. Neither expense nor effort will be spared to give value and variety to its contents. Articles will be illustrated with well executed engravings whenever they will add to the interest or assist to explanation.

Its extensive circulation and frequency of issue make it one of the BEST DENTAL ADVERTISING MEDIUMS in the United States.

All subscriptions must begin with January or July.

Local or special notices, five lines or less, will be inserted for $3 each insertion; over five lines, 50 cts. per line.

All advertising bills payable in advance, or at furthest, after the issue of the first number containing the advertisement. All transient advertisements to be paid for in advance.

☞CONTRIBUTIONS SOLICITED.

Exclusively Editorial Communications should be addressed to the Editor.

The latest number of the REGISTER may be ordered with or without other goods at any time, and all letters should be addressed to

SAM'L A. CROCKER & CO., Ohio Dental and Surgical Depot, Cincinnati, O.

